# Let bees be bees: Review of Seeley, T. D., 2019. The Lives of Bees: The Untold Story of the Honey Bee in the Wild. Princeton University Press, Princeton, NJ, 376 pp, $29,95, ISBN 9780691166766

**DOI:** 10.1186/s12052-020-00131-x

**Published:** 2020-07-29

**Authors:** Norman A. Johnson

**Affiliations:** Department of Biology, University of Massachusetts- Amherst, Amherst, MA 01003 USA

**Keywords:** Bees, Honey bees, Natural history, Reproductive biology

## Abstract

Thomas Seeley reviews a half century of research, mostly conducted by himself and his colleagues on the ecology, evolution, and natural history of wild honey bees. He provides insights into how bees reproduce, how they forage, and how they defend the colony. The final chapter, “Darwinian beekeeping” discusses how ecological and evolutionary principles can be incorporated into the practice of beekeeping.

Thomas Seeley, bee enthusiast and Horace White Professor of Biology at Cornell University has written a wonderful book, *The Lives of Bees: The Untold Story of the Honey Bee in the Wild*. This book explores the natural history, ecology, and evolution of honey bees (*Apis meliferia*). It primarily focuses on studies Seeley and his colleagues have done with wild bees around Ithaca, New York, where Seeley lives. Seeley who has been enraptured by bees since 1963, presents an authoritative and engaging account of why bees still fascinate him. The book should be of interests to students, educators, and professional biologists.

Seeley refers to honey bees as “honey bees”. My spellchecker wants to call them “honeybees”. I was not sure of the correct terminology, but I assumed that Seeley was likely correct. After some digging, I found out not only that Seeley was correct but also why. In his classic *Anatomy of the Honey Bee*, Snodgress (1956, P. vii) notes:

“Regardless of dictionaries, we have in entomology a rule for insect common names that can be followed. It says: If the insect is what the name implies, write the two words separately; otherwise run them together. Thus we have such names as house fly, blow fly, and robber fly contrasted with dragonfly, caddisfly, and butterfly, because the latter are not flies, just as an aphislion is not a lion and a silverfish is not a fish. The honey bee is an insect and preeminently a bee; ‘honeybee’ is equivalent to ‘Johnsmith’.”

In honey bees, the reproductive individual is the colony. Reproduction is limited to just the queen; except under very rare circumstances, female workers do not reproduce. Seeley says that we should consider the honey bee colony to be much like an apple tree with respect to their reproductive biology. Both are functionally hermaphrodites: the females and males of an apple tree are the seeds and pollen, respectively; while those of the honey bee colony are the queen and the drones. Females in both are enclosed in a protective covering: the delicious apple and the swarm of workers that surround the queen. In contrast to the protected female part, males in both are cheap and exposed. Males in both are produced in much greater quantities than the females.

Continuing the discussion of honey bee reproductive biology, Seeley addresses reproductive allocation. Evolutionary theory predicts that organisms will allocate resources equally to males and females (Hardy 2002). Do honey bee colonies follow this rule? Seeley first reminds us that the total female reproductive investment includes not just the queen, but also the workers in the surrounding swarm. He then presents work he and colleagues conducted determining the mass of male and female reproductive investment. They found that the total mass of the queens and workers in the swarm is 391 g (about 14 oz). For drones, the figure was not too much different—332 g (not quite 12 oz). Accordingly, the bees appear to follow the rules, investing comparable amounts in male and female reproduction.

Honey bees are distinct from most other bees. Nearly every other bee lives on an annual cycle. They reproduce and gather enough food to provide for their young, but then they die off, with the young persisting in the winter in a suspended amination state (diapause) until conditions permit them to develop (Embry 2018). Like the apple trees, honey bees are perennials. The colony goes on with active bees throughout the winter, even in areas of North America like Ithaca, New York where it can get and stay frigid for long stretches. The perennial lifestyle of honey bees necessitates that they gather large quantities of food to store for the winter. Seeley has shown that honey bee colonies in his neck of the woods consume an average of about 80 kg (176 lb) of honey and pollen combined each year! Seeley devotes considerable space to the mechanisms by which these bees go about collecting these mass quantities of food.

Being active over the long, cold winter also necessitates that bees thermoregulate. And honey bees excel at thermoregulation! The bees are essentially homotherms (warm blooded). Seeley walks us through the remarkable means by which bees alter their behavior upon temperature changes. The choice of nest is also important: as those living in cold climates can appreciate, lots of insulation cuts down on the heating costs. Honey bees also have several mechanisms by which they can cool off if it gets too hot during the summer. Among these is using water for evaporative cooling.

Seeley considers bees to be semidomesticated, instead of domesticated. He argues, “The human-animal relationship for honey bees is fundamentally different from that of cattle, chickens, horses, and other farm animals. In all of these species, selection is steered almost entirely by human hands for life…” (P. 80). In contrast, most of the selection on bees is natural selection, not imposed by human hands. Until about a century ago, we have not been able to systematically control the reproductive biology of bees. There are ecotypes of bees, but no breeds of bees, as in dogs. We have gotten bees to do our bidding more by changing their environment than by changing their genes. Some of these changes, such as the close spacing of bees in commercial beekeeping, have had unintended consequences. Close spacing appears to increase the risk of pathogens and parasites.

Honey bees are not native to the Americas; Bees collected in central New York State are genetically mainly from the southern European subspecies that came to America starting in the latter half of the 19th century. A sizeable, but minority, proportion comes from the northern European subspecies that came over with the first waves of Europeans. The overall genetic composition of the wild bees in the area had not changed much since the 1970s. Seeley and his group were able to reach these conclusions because Seeley had collected bees from areas near Cornell in 1977 and had deposited those bees in the Cornell University Insect Collection. Then his group collected bees more recently and sequenced both the museum and the more recent samples (Mikheyev et al. 2015). This study illustrates the value of insect collections and natural history museums in general for evolutionary biology research (Holmes et al. 2016).

This same study (Mikheyev et al. 2015) also provided insight into the impacts of an invasion of mites (*Varroa destructor*) on wild bees in the area. These mites had invaded the area and caused havoc on wild and commercial bees alike in between the times of the first and subsequent samples of bees. The post-mite sample showed much reduced genetic diversity in the maternally-inherited mitochondrial DNA, but little change in the diversity at the nuclear genome, This difference is likely because bees are outcrossing and each of the queens mating with many males. The study also found the signatures of selection on several genes–notably AmDOP3, which is linked to hygienic behavior. Perhaps further study of this gene will provide insight into engineering bees that are resistant to the mites.

Seeley concludes the book with a chapter on using insights from the ecology and evolutionary biology of bees to have beekeeping that would generate more healthy bees. He calls this Darwinian beekeeping. He suggests using bees that are adapted to the local environment; for example, bees from Georgia probably would not do that well in Ithaca, New York. He also suggests spacing out bee colonies to limit the spread of mites and disease. Just as we have been social distancing to limit the spread of the coronavirus, wild bees appear to engage in social distancing. Other recommendations include using smaller, and well-insulated hives. In summary, Darwinian beekeeping entails letting bees be bees.

## Data Availability

Not applicable.
